# A Case of *Ignatzschineria indica* Bacteremia following Maggot Colonization

**DOI:** 10.1155/2017/3698124

**Published:** 2017-11-02

**Authors:** Hugh Muse, Rachel L. Jenkins, Meredith B. Oliver, Soomin Kim, Richard L. Grantier, Bharat K. Malhotra, Jason J. Parham, Kayla R. Stover

**Affiliations:** ^1^University of Mississippi School of Medicine-Internal Medicine, Jackson, MS 39216, USA; ^2^University of Mississippi School of Pharmacy, Jackson, MS 39216, USA; ^3^St. Dominic-Jackson Memorial Hospital, Jackson, MS 39216, USA; ^4^University of Mississippi School of Medicine-Emergency Medicine, Jackson, MS 39216, USA; ^5^Department of Medicine-Hospital Medicine, University of Mississippi Medical Center, Jackson, MS 39216, USA; ^6^Department of Medicine-Infectious Diseases, Antimicrobial Stewardship Program, University of Mississippi Medical Center, Jackson, MS 39216, USA

## Abstract

*Ignatzschineria indica* is a Gram-negative bacterium that is commonly associated with the larvae of flesh flies. *I. indica* is difficult to isolate in routine laboratory procedures but has been associated with neglected wounds infested with maggots, fever, elevated white blood count and C-reactive protein, and polymicrobial culture results. Other specific hematological/immunological changes are not known. We present a case of *I. indica* bacteremia and polymicrobial osteomyelitis resulting from infected decubitus ulcers. The patient improved after treatment with cefepime followed by levofloxacin.

## 1. Introduction

*Ignatzschineria indica* is a Gram-negative aerobic nonmotile, non-spore-forming rod bacterium that belongs to the family Xanthomonadaceae and the class Gammaproteobacteria [1]. The *Ignatzschineria* genus is composed of three species (*I. indica*, *I. larvae*, and *I. ureiclastica*) which are common isolates from the larvae of the parasitic flesh fly, *Wohlfahrtia magnifica* [2]. The genus *Ignatzschineria* was renamed from *Schineria* in 2007 to honor Ignatz Rudolph Schiner, the entomologist who first described *Wohlfahrtia magnifica* [3]. More recent reports suggest that *Ignatzschineria* is not exclusively associated with *Wohlfahrtia* species and may be transmitted by other flesh flies as well [2]. Although *Ignatzschineria* sp. are generally difficult to isolate in routine laboratory procedures, they can be identified by 16S rRNA gene amplification and sequencing [1]. Human infections with pathogenic *I. indica* are uncommon and have only been described in case reports. When pathogenic, *I. indica* is relatively susceptible and can often be eradicated with a short course of oral antibiotics [2]. Here, we describe a case of *I. indica* bacteremia in a man with decubitus ulcers infested with maggots.

## 2. Case Report

A 46-year-old African American male (108.9 kg) with a past medical history of hypertension, stage 2 chronic kidney disease, and gout was brought to the emergency department by paramedics after being found unresponsive and hypotensive at home. The spouse of the patient called paramedics because the patient's breaths were shallow, and he was unresponsive. Chest compressions were initiated because paramedics could not feel a pulse. The patient was then intubated, and intravenous fluids and dopamine were given for hypotension.

**Figure 1:**
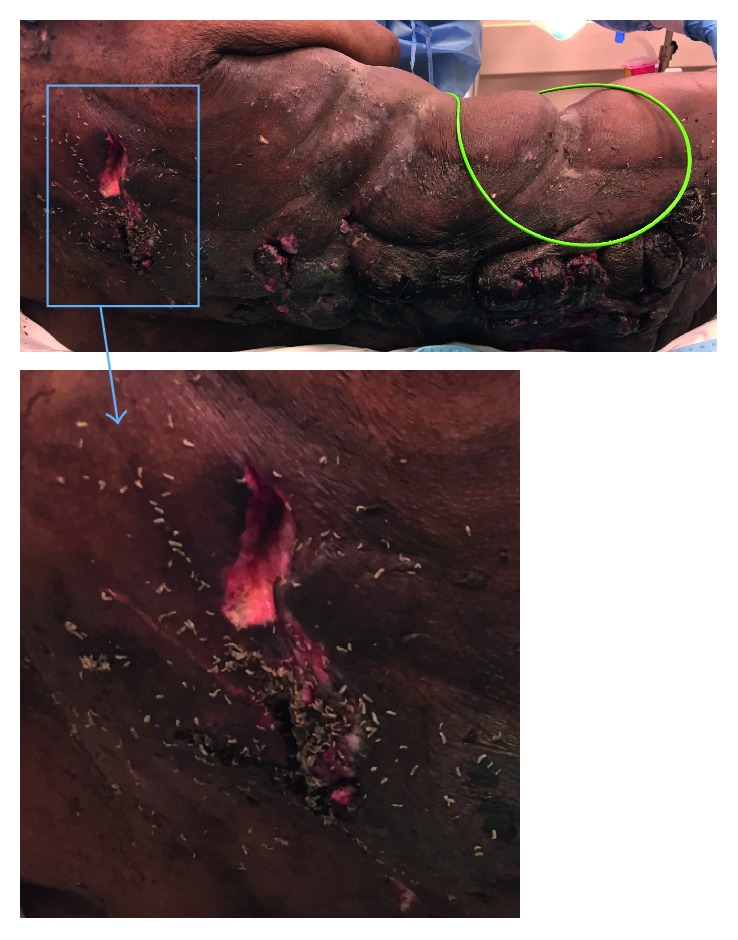
Our patient presented with neglected back wounds infested with maggots that ultimately may have been the source of *Ignatzschineria indica* in the bloodstream.

On arrival to the emergency department, the patient was found to be hypotensive and hypothermic with a blood pressure of 63/36 mmHg and a temperature of 30.9°C (87.6°F). Other vital signs included heart rate of 70 beats per minute, respiratory rate of 16 breaths per minute (intubated), and oxygen saturation of 99%. Physical exam was significant for multiple laceration-type lesions that were infested with maggots along the back covering the thoracic, lumbar, and sacral levels (Figure 1). A stage 4 sacral decubitus ulcer and an ulceration of the left heel were also noted on exam. Reportedly, the patient was bedbound on his couch for 6–8 months due to significant pain attributed to gout along with depression.

The patient was found to have lactic acidosis, hyperkalemia, leukocytosis, significant anemia, and acute kidney injury (Table 1). Urinalysis was positive for leukocyte esterase and had > 100 white blood cells per high power field (40x objective). Blood and urine cultures were obtained, and the patient was given intravenous fluid, norepinephrine, intermittent vancomycin 1750 mg IV, and piperacillin-tazobactam 3.375 g IV every 6 hours. The patient's hyperkalemia was treated medically; a blood transfusion was ordered for his anemia. The patient was stabilized with an improving blood pressure and was admitted to the medical intensive care unit for shock, attributed mostly to sepsis.

Initial blood cultures grew *Streptococcus gallolyticus*, *Streptococcus anginosus*, and an undetermined Gram-negative rod species. Urine cultures grew oxacillin-susceptible *Staphylococcus aureus*. Wound cultures from back grew *Proteus mirabilis* and diphtheroids. The undetermined Gram-negative was sent to Mayo Clinic Laboratories for further identification and was eventually identified as *Ignatzschineria indica*, susceptible to amikacin, aztreonam, cefepime, ceftazidime, ciprofloxacin, gentamicin, levofloxacin, meropenem, piperacillin-tazobactam, and tobramycin. On day 7, piperacillin-tazobactam was changed to cefepime 2 g IV every 12 hours and metronidazole 500 mg IV every 8 hours due to an increase in white blood cell count and worsening renal function.

A surgery team performed debridement of the back wounds and obtained cultures that grew *Escherichia coli* and *Proteus mirabilis*. A tissue sample from the sacrum was sent to pathology and was found to show acute osteomyelitis. Following surgery and antimicrobial therapy, the patient's clinical status improved. After susceptibilities for *I. indica* returned, vancomycin was discontinued on day 8, narrowing antimicrobial treatment to cefepime and metronidazole. On day 10, metronidazole was discontinued. After approximately 17 days of cefepime, treatment was changed to renally dosed oral levofloxacin (750 mg every 48 hours). The Infectious Diseases team recommended this treatment to be continued for 6 weeks from the initiation of cefepime in order to complete a course for osteomyelitis.

## 3. Literature Review

We searched PubMed with the keyword *Ignatzschineria* (17 results), *Ignatzschineria indica* (2 results), and *Schineria* (13 results) in June 2017 and found 7 case reports. In the first report in 2007, a homeless male presented with mild fever, increased C-reactive protein, normal white blood count, and wounds invaded by maggots [4]. His wound samples grew *Proteus mirabilis*, group A and group G streptococci, *Morganella* sp., *Bacteroides fragilis*, and *Candida albicans*. His blood culture was positive for a Gram-negative rod strain ADV4155.05, which was later identified as *Schineria* species. The strain was susceptible to beta-lactams, aminoglycosides, fluoroquinolones, erythromycin, rifampin, and colistin. Local debridement, bandaging, use of sulfadiazine, and ofloxacin 400 mg/day PO plus cefotaxime 6 g/day IV for 2 weeks resulted in clinical improvement. The patient was discharged with ciprofloxacin 500 mg/day PO plus amoxicillin-clavulanic acid 3 g/day PO for 20 days.

In 2014, three cases of *Ignatzschineria indica*-associated myiasis were published [2]. In the first of these three reports, a homeless male presented with a painful left foot that had been injured in a motor vehicle accident 2 months prior. He had been unable to treat his wounds or change the dressings since the accident. Upon undressing the wound, maggots were visible in the wound and between the digits. He had elevated C-reactive protein and a normal white blood cell count. Empiric therapy was started with ampicillin-sulbactam 3 g IV every 6 hours and vancomycin 1.25 g IV every 12 hours, necrotic tissue was debrided, and the third digit was amputated. Blood cultures were positive for nonhemolytic Gram-negative short plump rods that produced a “yellowish” pigment on blood agar. The organism was identified as *Ignatzschineria* (*Schineria*) *indica*. The patient was started on cephalexin 500 mg PO three times daily, discharged on day 3, and lost to follow-up.

In the second of three cases, a male with chronic alcoholism and extremely poor hygiene was admitted with chronic nonhealing ulcers in the left heel with maggot infestation and foul-smelling purulent discharge [2]. He was treated empirically with piperacillin-tazobactam IV and clindamycin IV. Blood cultures from the day before admission from an outside hospital grew *Streptococcus pyogenes* and *Ignatzschineria indica*. The latter isolate was susceptible to amikacin, gentamicin, tobramycin, cefepime, aztreonam, ciprofloxacin, levofloxacin, ticarcillin-clavulanate, and meropenem. The patient received a below-the-knee amputation and was treated with ciprofloxacin 500 mg PO twice daily and vancomycin 1 g IV every 12 hours for 2 weeks after the surgery.

In the final of the three cases, a paraplegic male was admitted for complications from a previous gunshot wound, including nonhealing decubitus ulcers and multiple past hospital admissions for urinary tract infections [2]. His urine cultures had been positive for *Escherichia coli*, *Proteus mirabilis*, vancomycin-resistant *Enterococcus faecalis*, *Pseudomonas aeruginosa*, *Providencia stuartii*, and on one occasion, an unidentifiable Gram-negative rod, which was later identified as *Ignatzschineria indica*. It was susceptible to aztreonam, ceftriaxone, cefepime, gentamicin, meropenem, trimethoprim-sulfamethoxazole, and tobramycin. It was unclear whether the patient received any treatment.

In 2015, a case reported the association of necrotizing wounds colonized by maggots with *Ignatzschineria*-associated septicemia in a man found unconscious in a forest [5]. He presented with cardiorespiratory arrest, cyanosis of the extremities, a necrotic skin lesion on the right shoulder, and many maggots around the genital organs. The patient was treated empirically with ceftriaxone IV. Blood cultures revealed *Enterococcus faecalis*, *Enterobacter cloacae*, *Providencia stuartii*, *Corynebacterium* spp., and a Gram-negative bacillus that was later identified as *Ignatzschineria ureiclastica*. The *I. ureiclastica* isolate was susceptible to all beta-lactams, aminoglycosides, fluoroquinolones, colistin, and trimethoprim/sulfamethoxazole. Ten days after hospital admission, the patient was found dead in his bed from no apparent cause, despite recent clinical improvement.

In a similar case reported in 2016, a man with a history of alcohol and nicotine abuse and COPD was admitted after being found unconscious and hypoxemic in front of his house. Results showed an elevated white blood cell count, C-reactive protein, and serum glucose [6]. Physical examination revealed a wound between his first and second toe on his right foot in which a great number of maggots were present. He was initially treated with steroids, bronchodilators, and amoxicillin/clavulanic acid. Blood cultures drawn upon admission revealed a Gram-negative, oxidase-positive, aerobic, catalase-positive rod later identified as *Ignatzschineria* (species not known) that was susceptible to amoxicillin/clavulanic acid and ciprofloxacin. It was found to be beta-lactamase positive. The wound was treated with povidone-iodine and melolin dressings, and a two-week course of amoxicillin/clavulanic acid was completed with good clinical response.

Finally, a female with a history of hypertension and diabetes presented with intense abdominal pain, constipation, and an elevated white blood cell count [7]. Physical examination revealed a left side fungating breast mass, malodorous necrotic tissue on the left nipple, and a left axillary abscess. She was empirically started on vancomycin IV and piperacillin/tazobactam IV. Cultures from abscess fluid drawn five days after admission revealed Gram-negative rods identified as *Proteus penneri*, *Providencia stuartii*, and *Ignatzschineria indica* (susceptible to amikacin, aztreonam, ceftazidime, ciprofloxacin, imipenem, levofloxacin, meropenem, trimethoprim/sulfamethoxazole, and tobramycin). The patient was treated for 14 days with piperacillin/tazobactam, resulting in reduced leukocytosis. She was discharged and referred to hematology/oncology.

## 4. Discussion

Over the past 10 years, there have been a handful of cases reported where *Ignatzschineria indica* was considered to be pathogenic [2, 4–7]. There are several similarities between case reports, predominantly wound infestation with maggots. This is not unexpected, as *Ignatzschineria* may be transmitted by multiple fly species, including the parasitic flesh fly, as described above [1, 2].

Similar to the cases presented by Roudiere, Barker, Brun, and Heddema, our patient was a male with poor hygiene who presented with a neglected wound infested with maggots, fever, elevated white blood count, and elevated C-reactive protein. As in five of the seven reported cases, our patient had polymicrobial culture results. In five cases, *I. indica* that was fairly susceptible to antibiotics was grown from blood cultures, similar to our patient.

Our patient received a longer course of antibiotic coverage compared to the other reported cases due to the evidence of bone involvement on pathology. Each patient presented with a varying degree of severity, resulting in differing outcomes.  Table 2 gives a summary of the cases, along with the prescribed treatments.

This compilation of cases demonstrates the need for clinicians to be aware of possible *Ignatzschineria* infection in patients presenting with poor hygiene and presence of maggots. Medical staff should consider collecting samples of maggots from these infested wounds for further evaluation by professional entomologists in order to better understand the transmission of *I. indica*.

## Figures and Tables

**Table 1:** Pertinent laboratory results.

Lab	Result
Bicarbonate (point of care)	8 mmol/L
Hemoglobin	4 g/dL
Lactate (point of care)	>16 mmol/L
pH (arterial blood gas)	7.01
Potassium (point of care)	6.9 mmol/L
Serum creatinine	3.1 mg/dL
White blood count	64,300/mL (84% neutrophils)

**Table 2:** Reported cases of *Ignatzschineria indica* as a human pathogen.

Reference	Year published	Patient sex	Patient age (yrs)	Geographical region/location	Culture source	Treatment	Outcome
Roudiere et al. [4]	2007	M	39	Montpellier, France	Blood	Ofloxacin plus cefotaxime then ciprofloxacin plus amoxicillin-clavulanate	Discharged to addiction treatment center

Barker et al. [2]	2014	M	64	Kentucky, United States	Blood	Ampicillin-sulbactam plus vancomycin then cephalexin	Discharged, lost to follow-up

Barker et al. [2]	2014	M	67	South Dakota, United States	Blood	Piperacillin-tazobactam plus clindamycin then ciprofloxacin and vancomycin	Discharged

Barker et al. [2]	2014	M	26	Texas, United States	Urine	Data unavailable	Data unavailable

Brun et al. [5]	2015	M	69	Loire Valley, France	Blood	Ceftriaxone	Death

Heddema et al. [6]	2016	M	71	Sittard-Geleen, Netherlands	Blood	Amoxicillin-clavulanate	Discharged, good clinical response

Mejias et al. [7]	2016	F	76	Pennsylvania, United States	Breast abscess	Vancomycin plus piperacillin-tazobactam then piperacillin-tazobactam	Discharged
